# Special Issue Introduction: Inherited Retinal Disease: Novel Candidate Genes, Genotype–Phenotype Correlations, and Inheritance Models

**DOI:** 10.3390/genes9040215

**Published:** 2018-04-14

**Authors:** Frans P. M. Cremers, Camiel J. F. Boon, Kinga Bujakowska, Christina Zeitz

**Affiliations:** 1 Department of Human Genetics and Donders Institute for Brain, Cognition and Behaviour, Radboud University Medical Center, P.O. Box 9101, 6500 HB Nijmegen, The Netherlands; 2 Department of Ophthalmology, Leiden University Medical Center, P.O. Box 9600, 2300 RC Leiden, The Netherlands; C.J.F.Boon@lumc.nl; 3 Academic Medical Center, Department of Ophthalmology, Meibergdreef 9, 1105 AZ Amsterdam, The Netherlands; 4 Ocular Genomics Institute, Massachusetts Eye and Ear Infirmary, Harvard Medical School, Boston, MA 02114, USA; Kinga_Bujakowska@MEEI.HARVARD.EDU; 5 Sorbonne Université, INSERM, CNRS, Institut de la Vision, Department of Genetics, 17 rue Moreau, 75012 Paris, France

Inherited retinal diseases (IRDs) are genetically and clinically heterogeneous disorders. Together, they have an estimated incidence of 1:2000 and thereby are the leading cause of vision loss in persons between 15 and 45 years of age [1,2,3]. IRDs can be clinically classified based on disease progression and the retinal cell types that are primarily involved in disease pathogenesis. They can be stationary, as for instance observed in most cases with congenital stationary night blindness (CSNB) and achromatopsia (ACHM), or progressive, such as in retinitis pigmentosa (RP), which is basically a rod-cone dystrophy, but also in cone-rod dystrophy (CRD) and Stargardt disease (STGD1). A second classification is based on the primary dysfunction or degeneration of the rod or cone photoreceptor cells. We can distinguish CSNB, which represents a dysfunction of retinal signaling from rods and cones to bipolar cells [4], and ACHM, or color blindness [5], in which one or more of the three types of cone cells are dysfunctional. In persons with CRD and STGD1, cones are affected first, followed by the degeneration of rods. This means that affected individuals initially experience central vision defects, which expand towards the mid-periphery. In persons with RP, this is the converse: initial clinical symptoms are night blindness and tunnel vision due to rod degeneration; central vision can also become impaired with progressive disease when cones also degenerate, eventually leading to legal blindness. Late in the disease process, both cones and rods are affected in CRD, RP, and STGD1, which make it difficult to come to a clear diagnosis. The most severe form of IRD is Leber congenital amaurosis (LCA), in which not only cones and rods of the neural retina can be affected simultaneously, but in which the retinal pigment epithelium (RPE) can also be primarily involved [6,7].

A few phenotypes, such as choroideremia (CHM) and STGD1, are caused by mutations in single genes, *CHM* and *ABCA4*, respectively [8,9]. In most of the IRDs, mutations in many different genes can cause very similar phenotypes. For example, mutations in 84 different genes underlie RP [10], 33 genes are implicated in cone dystrophy (CD)/CRD, 20 genes are involved in macular dystrophies (MD), 15 genes are involved in CSNB, and 9 genes are mutated in familial exudative vitreoretinopathy (FEVR) (Figure 1) [11]. 

Different variants in one gene can cause autosomal recessive (ar) or dominant (ad) retinal dystrophies (RDs), as exemplified by *GUCY2D* in which ad variants result in CRD and ar variants cause LCA [12,13]. Similarly, variants in rhodopsin (*RHO*) and *RP1* can be involved inadRP and arRP [14,15,16,17]. Although many arRDs are caused by mutations that result in the absence of functional protein, there are also several examples in which there is residual protein activity. Different combinations of mutations in some genes thereby can be associated with IRDs with different severity. For example, two null alleles in *ABCA4* result in early-onset CRD, whereas combinations of severe and mild variants result in intermediate or late-onset STGD1 [9,18,19,20,21]. Different combinations of mutations in the same gene can also cause syndromic and non-syndromic forms of arRDs. Some *USH2A* mutations either cause non-syndromic arRP or Usher syndrome type 2 [22,23]. Mutations in Bardet–Biedl syndrome (BBS)-associated genes, such as *BBS1*, can also be found in non-syndromic arRP [24,25,26], and *CEP290* variants underlie LCA, Senior–Løken syndrome, Joubert syndrome, or Meckel–Gruber syndrome [27,28,29,30]. Finally, bi-allelic null mutations in some IRD-associated genes may be lethal. The far majority of LCA cases carrying *NMNAT1* variants carry one hypomorphic variant and one null allele [31,32,33,34,35], and it was hypothesized that two *NMNAT1* null alleles could be lethal [35] or are associated with syndromic IRD.

The first retinal disease-associated gene identified was the ornithine aminotransferase (*OAT*) gene involved in gyrate atrophy. Reduced ornithine aminotransferase activity was measured in a patient’s cells in 1977 [36], and in 1988, the *OAT* gene was cloned, and the first mutation was identified [37]. Two years later, the second and third IRD-associated genes were identified. Mutations in the *RHO* gene, encoding the rod-specific light-sensitive chromophore, were identified in patients with adRP using a candidate gene approach [14] after linkage analysis in a large Irish adRP family had pointed towards a genomic region encompassing this gene [38]. In the same year, the *CHM* gene was identified using a positional cloning approach by mapping deletions in patients with syndromic and non-syndromic choroideremia [8]. 

The candidate gene approach (i.e., the search for IRD-associated variants in genes encoding proteins with known crucial functions in the retina) has been very successful. Similarly, comparing phenotypes of existing animal models with a known gene defect and subsequent screening of the respective candidate gene has identified many genes underlying IRD [4]. The identification of IRD-associated genes through their genomic position (i.e., positional cloning) as determined by linkage analysis has been used effectively, though this generally requires the availability of large families or a large set of families in which the same locus is involved. Linkage studies can be performed using microarrays that test thousands of single nucleotide polymorphisms (SNPs) spread across the genome. SNP microarrays have also proven very valuable for homozygosity and identity-by-descent (IBD) mapping of recessive disease genes, not only in consanguineous families [27,39], but also in small families and single patients of non-consanguineous marriages [40,41]. We are witnessing a new era in disease gene identification with the introduction of next-generation sequencing, allowing the analysis of all genes implicated in IRD [42] in a defined linkage interval, all exons in the genome (whole exome sequencing (WES)) [43,44,45,46], or even the entire genomic sequence (whole genome sequencing (WGS)) [47]. This also brings new challenges, such as data analysis and interpretation of genomic variants. Given the huge number of variants present in a patient’s genome, positional information on where the causative gene may be localized (e.g., by linkage analysis and/or homozygosity mapping) remains very helpful to pinpoint the genetic defect. Employing WGS, thousands of rare single nucleotide variants (SNVs) and structural variations (SVs) are found in every individual, and it remains very challenging to identify the causal variant(s). A functional read-out is required to identify the culprit variant(s). Gene-specific mRNA analysis or genome-wide mRNA analysis (transcriptome analysis) may identify quantitative or structural defects in mRNAs.

Whole exome sequencing and gene-panel sequencing analysis genetically solve 55–60% of these cases [42,48,49,50]. The hidden genetic variations may be unrecognized SVs and deep-intronic variations, which can be identified by WGS or gene-specific locus sequencing. Copy number variations (CNV)s can explain up to 18% of previously unsolved cases [51,52]. 

Receiving a molecular diagnosis becomes increasingly important with the development of (gene) therapy for IRDs. Up to 10 years ago, it was not possible to slow down, stabilize, or treat the vision impairment in patients with IRDs. This changed for a small group of patients with *RPE65* mutations, as gene augmentation was successfully and safely applied through subretinal injections of recombinant adeno-associated viruses (rAAVs) in Phase 1/2 trials [53,54,55]. Recombinant adeno-associated viruses transduce the RPE cells, upon which the viruses are shuttled to the nucleus, and the rAAV vector remains a stable extrachromosomal element. In the meantime, many more patients have been treated in two centers in Philadelphia and one in London. Vision improvement was variable and, in general, modest and appears to be more effective in younger patients. A Phase 3 trial was conducted in one center using an improved rAAV vector, which resulted in increased subjective and objective vision in the treated eye versus the untreated eye [56]. Gene therapy for *RPE65*—associated with LCA or RP—in the form of Luxturna is now an approved treatment in the United States (U.S. Food and Drug Administration STN: 125610). Gene augmentation targeting photoreceptors and the RPE was also successfully performed in a Phase 1/2 trial in choroideremia patients [57,58]. In addition, an oral 9-*cis* retinoid supplementation therapy seems effective in patients with *RPE65* and *LRAT* mutations [59,60]. Several therapies that will be developed in the next years will be gene-, or even mutation-specific, emphasizing the importance for patients to receive a molecular diagnosis. An overview of all ongoing gene therapy trials can be found on the internet [61].

Proving the involvement of a gene defect in an IRD and often a definite genetic diagnosis are dependent on modeling of the identified mutation(s) in animals or in ex vivo assays. Defects in several genes were previously found as naturally occurring or were modeled in rodents, zebrafish, and Drosophila [62] (and references therein). Human mutations have also been modeled by over-expression of wild-type and mutant proteins with a presumed dominant effect in cell culture [63]. More sophisticated approaches have been developed to study potential splicing alterations or hypomorphic alleles [64,65]. Some assays can be performed directly in cells available from patients [27]. However, in the absence of patients’ somatic cells that express the gene of interest, robust in vitro RNA splice assays can be set up for every human gene. In case retina-specific splice defects could play a role, photoreceptor precursor cells can be derived from induced pluripotent stem cells generated from blood cells or fibroblasts [66,67,68,69,70,71]. In addition to the new functional assays, the recent entrance of CRISPR/Cas opens great opportunities for IRD research, as it enables a more efficient introduction of specific mutations into animal models or cells [72]. This technology also offers a new therapeutic potential [73]. 

Only a few examples of digenic inheritance and modifier genes for IRDs have been reported [74,75,76,77,78]. Nevertheless, there are many examples of significant differences between phenotypes (e.g., age at onset) in IRD cases that carry the same mutation(s), both within and between families. Reduced penetrance of variants might explain several autosomal dominant conditions, but as yet we have few clues regarding the genetic and possibly non-genetic modifiers. To study the mechanism of variable expression and non-penetrance, large case/control cohorts and genome-wide analysis techniques, such as WES and WGS, are required. 

What are the future challenges in IRD research and diagnostics?

1. How can we determine causality for ultra-rare mutations in novel candidate genes? Apart from in silico tools that predict the potential causality of rare variants, we need to share our findings in a global manner. Tools for this are GeneMatcher [79,80] and intense collaborations, such as the European Retinal Disease Consortium (ERDC) [81].
2. How can we identify and functionally test non-coding variants?
3. To fully understand genotype–phenotype correlations, what are the effects of coding variants on RNA splicing and protein function?
4. Can we begin to understand phenotypic differences (and non-penetrance) of persons with the same mutations or the same types of mutations due to *cis* and *trans* modifiers or digenic inheritance?

With this special issue of the journal *Genes*, we address all the challenges mentioned above, except for the identification and functional testing of non-coding variants. In the paper by Astuti et al. [82] several probands carrying ultra-rare defects are presented in 11 novel candidate IRD genes through a European collaboration. The high aggregate carrier frequency of autosomal recessive variants associated with retinal dystrophies (up to 15%) [83] in some families with multiple affected individuals can result in the identification of independently acting defects in different genes, as shown by Gustafson et al. [84]. Llavona et al. [85] report on allelic mRNA imbalances for selected IRD-associated genes, which is very relevant to understanding phenotypic differences between individuals carrying the same genetic defects. El Shamieh et al. [86] report on additional RP-associated mutations in *KIZ*, encoding a ciliary protein, and the need to establish retina organoids from patient-derived iPS cells to understand the effect of these mutations on ciliary structure. The remaining nine manuscripts deal with genotype–phenotype correlations. They range from very large genotyping studies (e.g., the Target5000 study by Dockery et al. [87]) to targeted genotyping studies in pericentral RP (Comander et al. [88]) and early-onset RP and LCA families (Di Iorio et al. [89] and Porto et al. [90]). Brandl et al. [91] studied two genes encoding homologous proteins (IMPG1 and IMPG2) that are mutated in vitelliform macular dystrophies. Gene-specific studies were reported by McGuigan et al. (*EYS—*arRP) [92], Roosing et al. (*CEP290—*oligocone trichromacy) [93], Tracewska-Siemiątkowska et al. (YARS—RP, deafness, agenesis of the corpus callosum, and liver disease) [94], and Littink et al. (NRL—enhanced S-cone syndrome) [95].

## Figures and Tables

**Figure 1:**
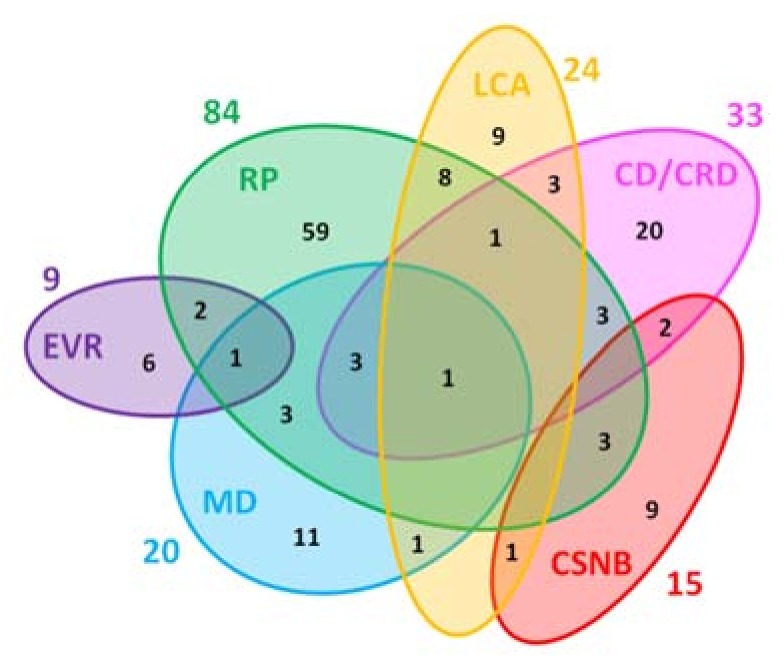
Genetic heterogeneity among the six major non-syndromic inherited retinal diseases (IRDs). Numbers outside of the ellipses correspond to the number of non-syndromic IRD genes responsible for the specific disease, while numbers within the ellipses correspond either to disease-specific genes or to genes mutated in two or more diseases. The non-redundant total of genes associated with these non-syndromic IRDs is 146. RP: retinitis pigmentosa; LCA: Leber congenital amaurosis; CD/CRD: cone dystrophy/cone-rod dystrophy; CSNB: congenital stationary night blindness; MD: macular dystrophy; EVR: exudative vitreoretinopathy.
